# In Vitro Inhibitory Effects of APINACA on Human Major Cytochrome P450, UDP-Glucuronosyltransferase Enzymes, and Drug Transporters

**DOI:** 10.3390/molecules24163000

**Published:** 2019-08-19

**Authors:** Sunjoo Kim, Won-Gu Choi, Mihwa Kwon, Sowon Lee, Yong-Yeon Cho, Joo Young Lee, Han Chang Kang, Im-Sook Song, Hye Suk Lee

**Affiliations:** 1 BK21 PLUS Team for Creative Leader Program for Pharmacomics-Based Future Pharmacy, College of Pharmacy, The Catholic University of Korea, Bucheon 14662, Korea; 2 College of Pharmacy and Research Institute of Pharmaceutical Sciences, Kyungpook National University, Daegu 41566, Korea

**Keywords:** APINACA, drug–drug interaction, cytochrome P450, uridine 5′-diphospho-glucuronosyltransferases, drug transporters

## Abstract

APINACA (known as AKB48, *N*-(1-adamantyl)-1-pentyl-1H-indazole-3-carboxamide), an indazole carboxamide synthetic cannabinoid, has been used worldwide as a new psychoactive substance. Drug abusers take various drugs concomitantly, and therefore, it is necessary to characterize the potential of APINACA-induced drug–drug interactions due to the modulation of drug-metabolizing enzymes and transporters. In this study, the inhibitory effects of APINACA on eight major human cytochrome P450s (CYPs) and six uridine 5′-diphospho-glucuronosyltransferases (UGTs) in human liver microsomes, as well as on the transport activities of six solute carrier transporters and two efflux transporters in transporter-overexpressed cells, were investigated. APINACA exhibited time-dependent inhibition of CYP3A4-mediated midazolam 1′-hydroxylation (*K_i_*, 4.5 µM; *k_inact_*, 0.04686 min^−1^) and noncompetitive inhibition of UGT1A9-mediated mycophenolic acid glucuronidation (*K_i_*, 5.9 µM). APINACA did not significantly inhibit the CYPs 1A2, 2A6, 2B6, 2C8/9/19, or 2D6 or the UGTs 1A1, 1A3, 1A4, 1A6, or 2B7 at concentrations up to 100 µM. APINACA did not significantly inhibit the transport activities of organic anion transporter (OAT)1, OAT3, organic anion transporting polypeptide (OATP)1B1, OATP1B3, organic cation transporter (OCT)1, OCT2, P-glycoprotein, or breast cancer resistance protein at concentrations up to 250 μM. These data suggest that APINACA can cause drug interactions in the clinic via the inhibition of CYP3A4 or UGT1A9 activities.

## 1. Introduction

Synthetic cannabinoids (SCs) are a type of new psychoactive substance that mimic Δ9-tetrahydrocannabinol (THC), the active component of cannabis, and typically bind to cannabinoid receptor type 1 or type 2 [1]. The misuse of SCs has increased worldwide, and 169 of these SCs have been monitored by the European Monitoring Centre for Drugs and Drug Addiction (EMCDDA) through the EU Early Warning System of December 2016.

**Figure 1:**
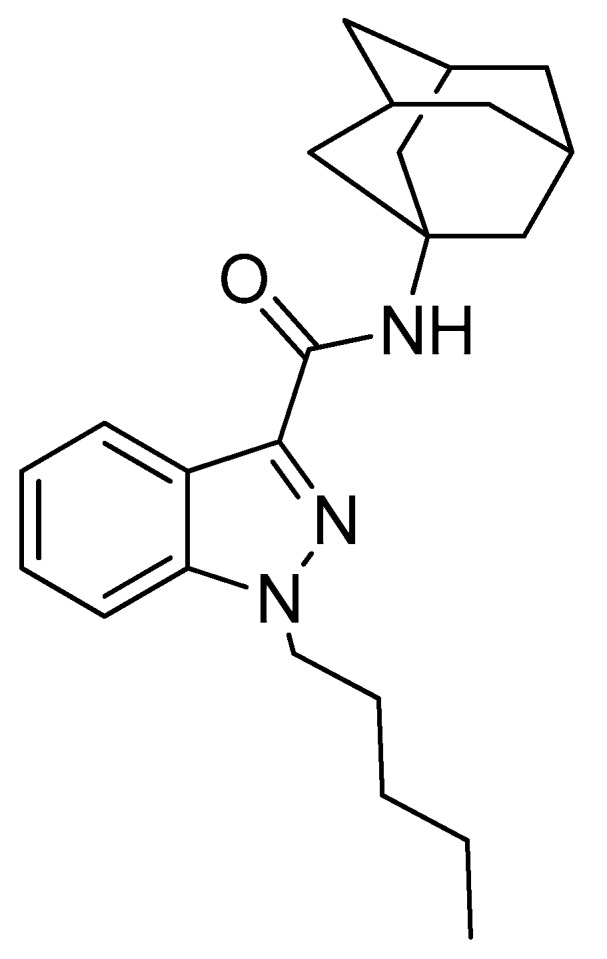
Chemical structure of APINACA (AKB48, *N*-(1-adamantyl)-1-pentyl-1H-indazole-3-carboxamide).

APINACA (known as AKB48, *N*-(1-adamantyl)-1-pentyl-1*H*-indazole-3-carboxamide) is an SC classified as an indazole carboxamide (Figure 1) [2]. APINACA was identified for the first time in Japanese herbal smoking blends in 2012 and has been categorized in Schedule I of the Controlled Substances Act by the US Drug Enforcement Administration since 2013. APINACA is extensively metabolized to 10 metabolites in vitro and in vivo via hydroxylation and oxidation at pentyl and adamantyl moieties by cytochrome P450 (CYP) enzymes, such as CYPs 1A2, 2C8, 2C9, 2C19, 2D6, and 3A4, and via carboxylation on the pentyl group by alcohol dehydrogenase/acetaldehyde dehydrogenase [3,4,5,6,7].

Drugs not only are the substrates for drug-metabolizing enzymes, such as CYP and uridine 5′-diphospho-glucuronosyltransferase (UGT), but also may cause drug interactions with coadministered drugs and affect drug metabolism via the inhibition or induction of CYP and UGT enzymes [6,8,9]. Drugs are also the substrate for transporters that play crucial roles in the absorption and disposition of these drugs: therefore, transporter-mediated drug–drug interactions have become an important issue in drug-metabolizing enzyme-mediated drug interactions [10]. According to the guidelines of the US Food and Drug Administration and the International Transporter Consortium, it is necessary to evaluate the effects of new drug candidates on clinically important solute carrier transporters, including organic cation transporter (OCT)1, OCT2, organic anion transporter (OAT)1, OAT3, organic anion transporting polypeptide (OATP)1B1, and OATP1B3; and efflux transporters, such as P-glycoprotein (P-gp) and breast cancer resistance protein (BCRP), to predict transporter-mediated drug–drug interactions.

The concomitant uses of diverse drugs by drug abusers have been frequently reported [11,12,13], and therefore, it is necessary to investigate the inhibitory effects of abused drugs on major drug-metabolizing enzymes, such as CYPs and UGTs, and on clinically important drug transporters, such as solute carrier transporters and efflux transporters. The inhibitory effects of SCs, such as AM-2201 [14], MAM-2201 [15], EAM-2201 [16], JWH-019, STS-135, and UR-144 [17], and of phytocannabinoids, such as THC, cannabinol, and cannabidiol [18,19,20,21,22,23,24], on major human CYP and UGT enzyme activities in human liver microsomes or on recombinant CYP and UGT enzymes have been reported.

The effects of various abused drugs on transporters have been reported to predict transporter-mediated drug interactions: for example, buprenorphine, norbuprenorphine, ibogaine, methadone, and THC inhibited P-gp in HEK293-MDR1 cells, and buprenorphine, norbuprenorphine, and ibogaine inhibited BCRP in HEK293BCRP cells [25]. Phytocannabinoids (THC, cannabinol, and cannabidiol), JWH-200, and WIN-55,212-2 inhibited BCRP ATPase activity [26,27]. Diclofensine, glaucine, 2,5-dimethoxy-4-iodoamphetamine, *N*-isopropyl-1,2-diphenylethylamine, and *N*-(1-phenylcyclohexyl)-3-ethoxypropanamine stimulated similar ATPase activity to verapamil and sertraline [28]. Glaucine, JWH-200, mitragynine, and WIN-55,212-2 were shown not to be P-gp substrates, but P-gp inhibitors, in Caco-2 cells [29]. APINACA inhibited dopamine uptake in the human dopamine transporter overexpressed system, with a *K_i_* value of 4.6 µM [30].

However, no report exists concerning the in vitro and in vivo inhibitory effects of APINACA on major human drug-metabolizing enzymes, such as CYPs and UGTs, solute carrier transporters, and efflux transporters.

The purpose of this study was to investigate the in vitro inhibitory effects of APINACA on the activities of eight major human CYPs and six UGTs in ultrapooled human liver microsomes and on the transport activities of six solute carrier transporters and two efflux transporters in transporter-overexpressed cells to predict the potentials for APINACA-induced drug interactions.

## 2. Results

### 2.1. Inhibitory Effect of APINACA on CYP and UGT Enzymes in Human Liver Microsomes

**Figure 2:**
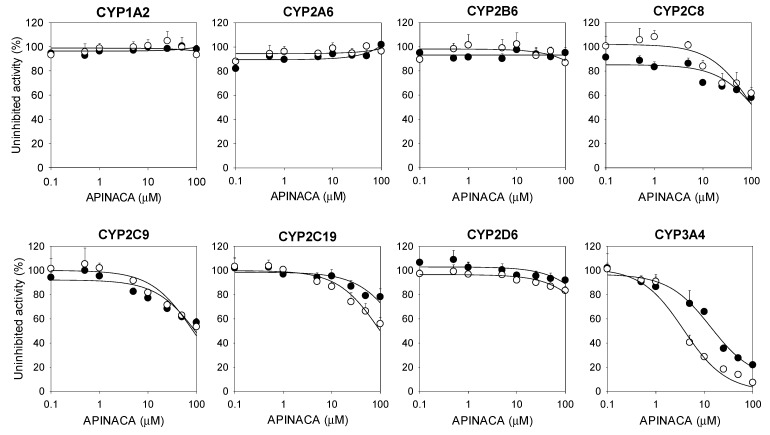
Inhibitory effect of APINACA on CYP1A2-mediated phenacetin *O*-deethylase, CYP2A6-mediated coumarin 7-hydroxylase, CYP2B6-mediated bupropion hydroxylase, CYP2C8-catalyzed amodiaquine *N*-deethylase, CYP2C9-catalyzed diclofenac 4′-hydroxylase, CYP2C19-mediated [*S*]-mephenytoin 4′-hydroxylase, CYP2D6-mediated bufuralol 1′-hydroxylase, and CYP3A4-mediated midazolam 1′-hydroxylase activities in ultrapooled human liver microsomes with (◯) and without (⬤) a 30-min preincubation in the presence of NADPH at 37 °C. The cocktail substrate concentrations used to assess the IC_50_ values were as follows: 50 μM phenacetin, 2.5 μM coumarin, 2.0 μM amodiaquine, 10 μM diclofenac, 100 μM [*S*]-mephenytoin, 5.0 μM bufuralol, and 2.5 μM midazolam. Inhibition of CYP2B6 was evaluated separately using 50 μM bupropion. The data are the means ± SD (*n* = 3).

The reversible and time-dependent inhibitory effects (IC_50_ values) of APINACA on eight major human CYP enzyme activities in ultrapooled human liver microsomes were evaluated using a liquid chromatography–tandem mass spectrometric (LC–MS/MS) assay and a cocktail of CYP substrates (Figure 2, Table 1). APINACA potently inhibited CYP3A4-catalyzed midazolam 1′-hydroxylation with an IC_50_ value of 16.9 μM in human liver microsomes, and 30-min preincubation of APINACA with ultrapooled human liver microsomes and reduced β-nicotinamide adenine dinucleotide phosphate (NADPH) resulted in ca. 4-fold lower IC_50_ value of CYP3A4-catalyzed midazolam 1′-hydroxylase activity (4.2 μM) than with no preincubation (Figure 2, Table 1), indicating that APINACA might be a time-dependent inhibitor of CYP3A4. APINACA did not significantly inhibit CYP1A2-mediated phenacetin *O*-deethylation, CYP2A6-mediated coumarin 7-hydroxylation, CYP2B6-mediated bupropion hydroxylation, CYP2C8-catalyzed amodiaquine *N*-deethylation, CYP2C9-mediated diclofenac 4′-hydroxylation, CYP2C19-mediated [*S*]-mephenytoin 4′-hydroxylation, and CYP2D6-mediated bufuralol 1′-hydroxylation activities (IC_50_ > 100 μM) in ultrapooled human liver microsomes with and without 30-min preincubation (Figure 2, Table 1). The inhibitory potentials of typical CYP inhibitors were also evaluated in ultrapooled human liver microsomes, as follows: α-naphthoflavone (IC_50_, 0.06 μM), sulfaphenazole (IC_50_, 0.8 μM), [*S*]-benzylnirvanol (IC_50_, 0.1 μM), quinidine (IC_50_, 0.6 μM), and ketoconazole (IC_50_, 0.09 μM) selectively inhibited CYP1A2, CYP2C9, CYP2C19, CYP2D6, and CYP3A4, respectively.

The data represent the average of three measurements.

**Figure 3:**
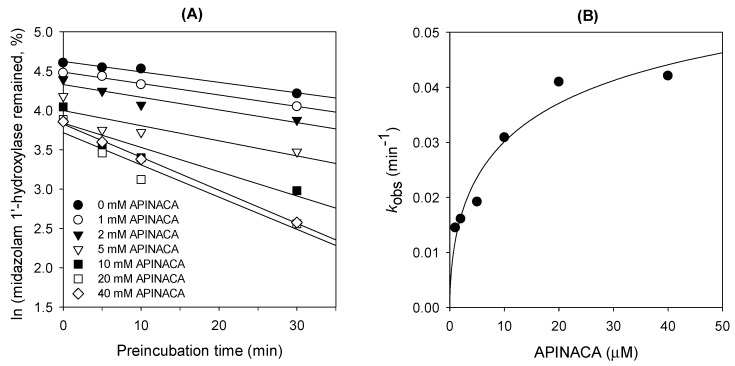
(**A**) Inactivation kinetics of 1′-hydroxymidazolam formation from midazolam in ultrapooled human liver microsomes using various concentrations of APINACA and (**B**) the relationship between the *k*_obs_ values and the APINACA concentrations to determine the *K_i_* and *k_inact_* values for the time-dependent inhibition of CYP3A4-mediated midazolam 1′-hydroxylation.

The *K_i_* and *k_inact_* values of APINACA for the time-dependent inhibition of CYP3A4-catalyzed midazolam 1′-hydroxylase were 4.5 μM and 0.04686 min^−1^, respectively (Figure 3).

**Figure 4:**
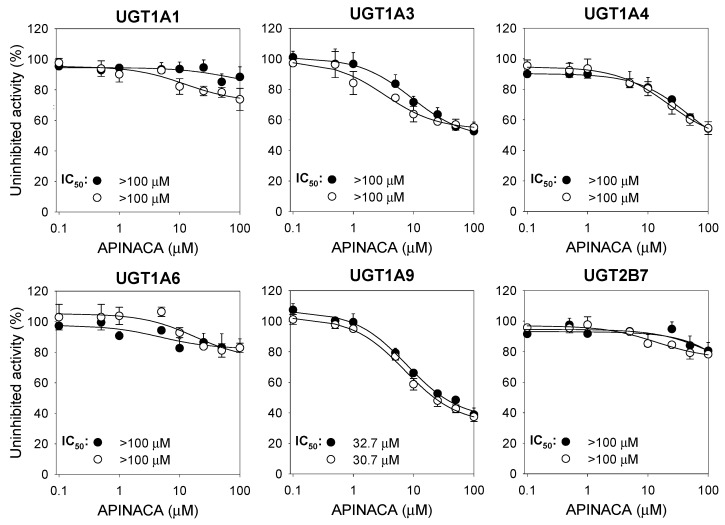
Inhibitory effect of APINACA on the metabolic activities of six uridine 5′-diphospho-glucuronosyltransferase (UGT) enzymes in pooled human liver microsomes with (◯) and without (⬤) a 30-min preincubation in the presence of uridine 5′-diphosphoglucuronoic acid (UDPGA) at 37 °C. The cocktail UGT substrate concentrations were as follows: 0.5 µM SN-38 for UGT1A1, 2 µM chenodeoxycholic acid for UGT1A3, 0.5 µM trifluoperazine for UGT1A4, 1 µM *N*-acetylserotonin for UGT1A6, 0.2 µM mycophenolic acid for UGT1A9, and 1 µM naloxone for UGT2B7. The data are the means ± SD (*n* = 3).

APINACA moderately inhibited UGT1A9-catalyzed mycophenolic acid glucuronidation with an IC_50_ value of 32.7 μM but negligibly inhibited UGT1A1-catalyzed SN-38 glucuronidation, UGT1A3-catalyzed chenodeoxycholic acid 24-acyl-β-glucuronidation, UGT1A4-catalyzed trifluoperazine *N*-glucuronidation, UGT1A6-catalyzed *N*-acetylserotonin glucuronidation, and UGT2B7-catalyzed naloxone 3-β-d-glucuronidation in human liver microsomes at 100 μM with and without 30 min preincubation (Figure 4). The inhibitory potentials of UGT inhibitors were also evaluated in ultrapooled human liver microsomes: atazanavir (IC_50_, 0.7 μM), glycyrrhetic acid (IC_50_, 4.2 μM), efavirenz (IC_50_, 2.0 μM), troglitazone (IC_50_, 6.0 μM), magnolol (IC_50_, 9.7 μM), and diclofenac (IC_50_, 29.4 μM) inhibited UGT1A1, UGT1A3, UGT1A4, UGT1A6, UGT1A9, and UGT2B7, respectively.

**Figure 5:**
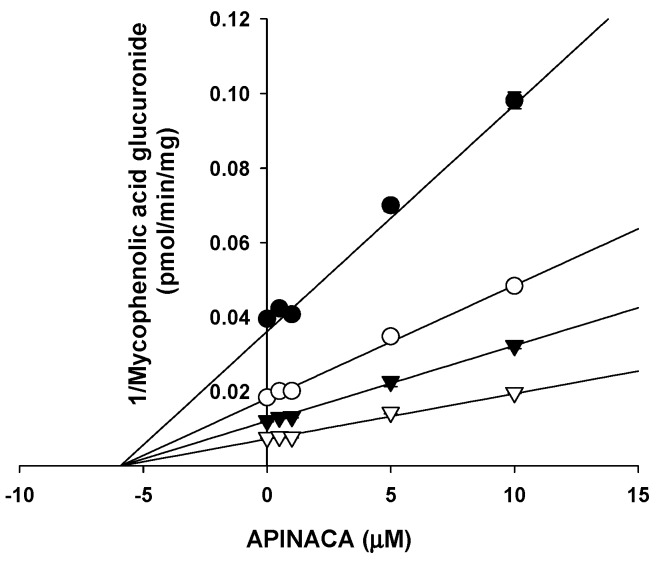
Representative Dixon plot for the inhibitory effect of APINACA on UGT1A9-catalyzed mycophenolic acid glucuronidation in ultrapooled human liver microsomes. Each symbol represents the concentration of mycophenolic acid: ⬤, 0.2 μM; ◯, 0.4 μM; ▼, 0.6 μM; ▽, 1 μM. The data are the means ± SD (*n* = 3).

Based on an enzyme kinetics study, APINACA noncompetitively inhibited UGT1A9-catalyzed mycophenolic acid glucuronidation with a *K_i_* value of 5.9 μM in human liver microsomes (Figure 5).

### 2.2. Inhibitory Effect of APINACA on Drug Transporters

The functionality of these transport systems was confirmed by the significantly greater transport rates in the probe substrates in HEK293 cells overexpressing solute carrier transporters or LLC-PK1 cells overexpressing efflux transporters than in mock cells (Table 2), consistent with our previous reports [31,32,33]. Using the same system, inhibitory potentials of typical inhibitors of uptake and efflux transporters were evaluated. Cimetidine inhibited OCT1 and OCT2 with IC_50_ values of 61.8 μM and 197.2 μM, respectively. Probenecid inhibited OAT1 and OAT3 with IC_50_ values of 7.56 μM and 4.13 μM, respectively. Rifampin inhibited OATP1B1 and OATP1B3 with IC_50_ values of 22.8 μM and 0.99 μM, respectively. Verapamil and sulfasalazine inhibited P-gp and BCRP with IC_50_ values of 5.61 μM and 0.39 μM, respectively.

**Figure 6:**
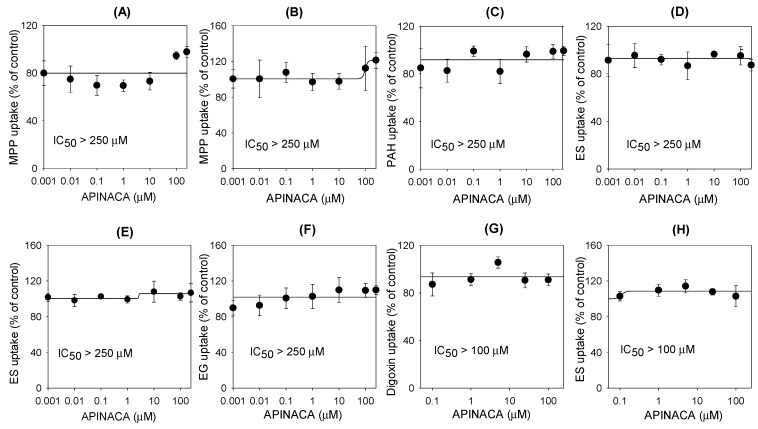
Inhibitory effect of APINACA on the transport of the probe substrates in (**A**) HEK293-OAT1, (**B**) HEK293-OAT3, (**C**) HEK293-OCT1, (**D**) HEK293-OCT3, (**E**) HEK293-OATP1B1, (**F**) HEK293-OATP1B3, (**G**) LLC-PK1-MDR1, and (**H**) LLC-PK1-BCRP cells. The concentrations and probe substrates were selected as follows: 0.1 μM [3-H]methyl-4-phenylpyridinium (for OCT1 and OCT2), 0.1 μM [3-H]para-aminohippuric acid (for OAT1), 0.1 μM [3-H]estrone-3-sulfate (for OAT3, OATP1B1, and BCRP), 0.1 μM [3-H]estradiol-17β-d-glucuronide (for OATP1B3), and 0.1 μM [3-H]digoxin (for P-glycoprotein (P-gp)). The data are the means ± SD (*n* = 3).

The inhibitory effects of APINACA on eight major transporters were evaluated using mammalian cells overexpressing OCT1, OCT2, OAT1, OAT3, OATP1B1, OATP1B3, P-gp, and BCRP. APINACA did not inhibit significantly the transport activities of OAT1, OAT3, OATP1B1, OATP1B3, OCT1, OCT2, P-gp, or BCRP in the concentration ranges tested (Figure 6).

## 3. Discussion

APINACA inhibited only CYP3A4-catalyzed midazolam 1′-hydroxylation in a preincubation time- and concentration-dependent manner in human liver microsomes and did not inhibit the other seven CYP enzymes (Figure 2 and Figure 3). The *K_i_* and *k_inact_* values of APINACA for CYP3A4-catalyzed midazolam 1′-hydroxylation in human liver microsomes were 4.5 μM and 0.04686 min^−1^, respectively (Figure 3B), and were comparable to those of EAM-2201, a halogenated naphthoylindole SC (*K_i_*, 4.1 μM; *k_inact_*, 0.0250 min^−1^) [16]. AM-2201 (*K*_i_, 4.0 µM) and MAM-2201 (*K*_i_, 5.4 µM), halogenated naphthoylindole SCs, showed competitive and noncompetitive inhibition of CYP3A4-catalyzed midazolam 1′-hydroxylation, respectively [14,15], and cannabidiol competitively inhibited CYP3A4-catalyzed diltiazem *N*-demethylation (*K*_i_, 6.14 µM) in human liver microsomes [23]. The inactivation efficiency (*k_inact_*/*K_i_*) value of APINACA for the time-dependent inhibition of CYP3A4 (10.4 mL/μmol/min) was comparable to that of verapamil (11.2 mL/μmol/min) and clarithromycin (2.8 mL/μmol/min), the causes of clinical CYP3A4-mediated drug interaction, in human liver microsomes [34]. This result has implications for the in vivo drug interaction potentials of APINACA with CYP3A4 substrates, such as atorvastatin, clarithromycin, cyclosporine, felodipine, lovastatin, midazolam, nifedipine, simvastatin, and MAM-2201 [15,35].

APINACA showed potent noncompetitive inhibition of UGT1A9-catalyzed mycophenolic acid glucuronidation, with a *K_i_* value of 5.9 μM (Figure 5). Cannabidiol inhibited UGT1A9-mediated ethanol glucuronidation (*K_i_*, 9.9 μM) in human liver microsomes [36]. Vandetanib (*K_i_*, 9.0 μM), canagliflozin (*K_i_*, 1.4–3.0 μM), dapagliflozin (*K_i_*, 11–15 μM), sorafenib (*K_i_*, 0.7 μM), and regorafenib (*K_i_*, 0.7 μM) inhibited recombinant and human liver microsomal UGT1A9 activities, resulting in clinically significant drug–drug interactions [37,38,39]. This result reveals that it is necessary to evaluate the in vivo drug interaction potentials of APINACA with UGT1A9 substrates, such as phenylbutazone, sulfinpyrazone, sorafenib, oxazepam, propranolol, and propofol [40,41].

On the other hand, APINACA poorly inhibited solute carrier transporters, such as OAT1, OAT3, OCT1, OCT2, OATP1B1, and OATP1B3; and efflux transporters, such as P-gp and BCRP, even though APINACA was treated at high concentrations (up to 250 μM), suggesting that APINACA has a low potential for drug interactions in association with these transporters. Therefore, APINACA exposure in human blood caused by APINACA abuse might not potentiate the transporter-mediated toxicity or adverse events of APINACA.

For the accurate prediction of APINACA-induced drug interaction potential in the clinic from in vitro data, information regarding APINACA pharmacokinetics in humans, including plasma concentrations, protein binding, tissue distribution, etc., is necessary. However, there has been no report on the absorption, distribution, and excretion of APINACA in humans and animals. In one study, APINACA was determined concomitantly with 5F-APINACA in blood samples of three “driving under the influence of drugs” cases, and its blood concentrations were 0.66–67.1 nM [42], which did not accurately reflect the maximum blood concentrations and the tissue concentrations of APINACA in the liver. Although the inhibition of CYP and UGT activities in vitro does not necessarily translate into drug interactions in clinical situations, it is necessary to evaluate the potential of in vivo pharmacokinetic drug–drug interactions via APINACA-induced inhibition of CYP3A4 and UGT1A9 activities in the clinic.

## 4. Materials and Methods

### 4.1. Materials

APINACA was obtained from Cayman Chemical Company (Ann Arbor, MI, USA). Acetaminophen, α-naphthoflavone, *N*-acetylserotonin, alamethicin, atazanavir, chenodeoxycholic acid, cimetidine, coumarin, 7-hydroxycoumarin, magnolol, midazolam, mycophenolic acid, naloxone, naloxone 3-β-d-glucuronide, NADPH, phenacetin, probenecid, quindine, rifampin, sulfaphenazole, sulfasalazine, trifluoperazine, Trizma base, uridine 5′-diphosphoglucuronoic acid (UDPGA), verapamil, sodium dodecyl sulfate (SDS), and Hank’s balanced salt solution (HBSS) were obtained from Sigma-Aldrich (St. Louis, MO, USA). ^13^C_2_,^15^N-acetaminophen; bufuralol; *N*-desethylamodiaquine; 1′-hydroxybufuralol; d_9_-1′-hydroxybufuralol; 4′-hydroxydiclofenac; 4′-hydroxymephenytoin; 1′-hydroxymidazolam; [*S*]-mephenytoin; Dulbecco’s Modified Eagle’s Medium (DMEM); medium 199; fetal bovine serum (FBS); collagen-coated 12-transwell plates; poly-D-lysine-coated 24-well plates; ultrapooled human liver microsomes (150 donors, mixed gender); LLC-PK1-MDR1 (LLC-PK1 cells stably expressing P-gp); LLC-PK1-mock cells; HEK293 cells transiently overexpressing OCT1, OCT2, OAT1, OAT3, OATP1B1, and OATP1B3 transporters (HEK293-OCT1, -OCT2, -OAT1, -OAT3, -OATP1B1, and -OATP1B3, respectively); and HEK293-mock cells were purchased from Corning Life Sciences (Woburn, MA, USA). LLC-PK1-BCRP (LLC-PK1 cells stably expressing BCRP) and LLC-PK1-mock cells were obtained from Dr. A.H. Schinkel (Netherlands Cancer Institute, Amsterdam, the Netherlands). In addition, [3-H]Methyl-4-phenylpyridinium ([3-H]MPP^+^, 2.9 TBq/mmol), [3-H]para-aminohippuric acid ([3-H]PAH, 0.13 TBq/mmol), [3-H]estrone-3-sulfate ([3-H]ES, 2.12 TBq/mmol), [3-H]estradiol-17β-d-glucuronide ([3-H]EG, 2.22 TBq/mmol), and [3-H]digoxin (1.103 TBq/mmol) were purchased from Perkin Elmer, Inc. (Boston, MA, USA). *N*-acetylserotonin β-d-glucuronide, chenodeoxycholic acid 24-acyl-β-glucuronide, diclofenac, efavirenz, ketoconazole, mycophenolic acid β-d-glucuronide, [*S*]-benzylnirvanol, troglitazone, and SN-38 glucuronide were obtained from Toronto Research Chemicals (Toronto, ON, Canada). SN-38 was obtained from Santa Cruz Biotechnology (Dallas, TX, USA). Glycyrrhetic acid was obtained from Tokyo Chemical Industry (Tokyo, Japan). Acetonitrile, methanol, and water (LC–MS grade) were obtained from Fisher Scientific Co. (Fair Lawn, NJ, USA). All other chemicals were of the highest quality available.

### 4.2. Inhibitory Effect of APINACA on Eight Major CYP Activities in Human Liver Microsomes

The inhibitory potentials (IC_50_ values) of APINACA and typical CYP inhibitors on CYP 1A2, 2A6, 2C8, 2C9, 2C19, 2D6, and 3A4 activities in pooled human liver microsomes were evaluated following our previous method using a cocktail of CYP substrates, followed by LC–MS/MS [43]. The incubation mixtures were prepared in total volumes of 100 μL as follows: 50 mM potassium phosphate buffer (pH 7.4), 1.0 mM NADPH, 10 mM MgCl_2_, ultrapooled human liver microsomes (0.2 mg/mL), various concentrations of APINACA (final concentrations of 0.1–100 μM) or typical CYP inhibitors, and a cocktail of seven CYP probe substrates (2.0 μM amodiaquine, 5 μM bufuralol, 2.5 μM coumarin, 10 μM diclofenac, 100 μM [*S*]-mephenytoin, 2.5 μM midazolam, and 50 μM phenacetin). After 3 min of preincubation at 37 °C, the reaction mixtures were incubated for 15 min at 37 °C with the addition of NADPH in a shaking water bath. The reaction was stopped by adding 100 μL of ice cold methanol containing internal standards (d_9_-1′-hydroxybufuralol for 1′-hydroxybufuralol, 4′-hydroxydiclofenac, 7-hydroxycoumarin, 1′-hydroxymidazolam, and 4′-hydroxymephenytoin; ^13^C_2_, ^15^N-acetaminophen for acetaminophen and *N*-desethylamodiaquine). The incubation mixtures were centrifuged at 13,000× *g* for 8 min at 4 °C, and 50 μL of each supernatant was diluted with 50 μL of water. Aliquots (5 μL) of the diluted supernatants were analyzed by LC–MS/MS. All assays were performed in triplicate, and the average values were used for subsequent calculations.

To evaluate the inhibitory effect of APINACA on CYP2B6-catalyzed bupropion hydroxylation, each incubation mixture in a total volume of 100 μL contained 50 mM potassium phosphate buffer (pH 7.4), 10 mM MgCl_2_, 0.2 mg/mL pooled human liver microsomes, 50 μM bupropion, and various concentrations of APINACA in methanol (final concentrations of 0.1–100 μM), according to our previous report [43]. After 3 min of preincubation at 37 °C, the reaction mixtures were incubated with the addition of NADPH in a shaking water bath for 15 min at 37 °C. The reaction was stopped by adding 100 μL of ice cold d_9_-1′-hydroxybufuralol (internal standard) in methanol. The mixtures were centrifuged at 13,000× *g* for 8 min at 4 °C. All incubations were performed in triplicate, and the average values were used for subsequent calculations.

To measure the time-dependent inhibition, human liver microsomes were preincubated with the various concentrations of APINACA (final concentrations of 0.1−100 μM) and NADPH for 30 min at 37 °C. Next, the reaction mixtures were incubated with a seven-CYP probe substrate cocktail or bupropion for 15 min at 37 °C. The control reaction was performed by adding methanol instead of the test compounds.

The LC-MS/MS system was comprised of an Agilent 6495 triple quadrupole mass spectrometer coupled with an Agilent 1290 Infinity system (Agilent Technologies, CA, USA). The column and autosampler temperatures were 40 °C and 4 °C, respectively.

The metabolites formed from the eight CYP substrates were simultaneously separated on an Atlantis dC18 (3 µm, 2.1 mm internal diameter × 100 mm; Waters Co., MA, USA) using a gradient elution of 5% methanol in 0.1% formic acid (mobile phase A) and 95% methanol in 0.1% formic acid (mobile phase B) at a flow rate of 0.3 mL/min: 15% mobile phase B for 1.5 min, 15% to 50% mobile phase B for 0.5 min, 50% to 95% mobile phase B for 2 min, 95% mobile phase B for 2 min, 95% to 15% mobile phase B for 0.1 min, and 15% mobile phase B for 3 min. The electrospray ionization (ESI) source settings in the positive ion mode were as follows: gas temperature, 200 °C; gas flow, 14 L/min; nebulizer, 40 psi; sheath gas temperature, 380 °C; sheath gas flow, 11 L/min; capillary voltage, 4500 V; and nozzle voltage, 500 V. Quantification of each metabolite was performed by the selected reaction monitoring mode: acetaminophen, *m/z* 152.1 → 110.1; 7-hydroxycoumarin, *m/z* 163.0 → 107.0; 4-hydroxybupropion, *m/z* 256.1 → 238.0; *N*-desethylamodiaquine, *m/z* 328.1 → 283.0; 4′-hydroxydiclofenac, *m/z* 312.0 → 231.0; 4′-hydroxymephenytoin, *m/z* 235.2 → 150.0; 1′-hydroxybufuralol, *m/z* 278.3 → 187.0; 1′-hydroxymidazolam, *m/z* 342.1 → 324.1; ^13^C_2_, ^15^N-acetaminophen, *m/z* 155.1 → 111.1; and d_9_-1′-hydroxybufuralol, *m/z* 287.0 → 187.0. The data were processed using Mass Hunter software (Agilent Technologies).

### 4.3. Inhibitory Effect of APINACA on Six Major UGT Activities

The inhibitory effect of APINACA and typical UGT inhibitors on UGT1A1, UGT1A3, UGT1A4, UGT1A6, UGT1A9, and UGT2B7 were evaluated following our previous method by LC–MS/MS after incubation of ultrapooled human liver microsomes with a cocktail of UGT substrates [44]. Each incubation mixture was prepared in a final volume of 100 μL as follows: ultrapooled human liver microsomes (0.2 mg/mL), 5 mM UDPGA, 10 mM magnesium chloride, alamethicin (25 μg/mL), 50 mM Tris buffer (pH 7.4), various concentrations of APINACA in methanol (final concentrations of 0.1–100 μM, methanol <0.5% (*v/v*)) or typical UGT inhibitors, and the UGT enzyme-specific substrates of the cocktail set (A set: 0.5 μM SN-38, 2 μM chenodeoxycholic acid, and 0.5 μM trifluoperazine; B set: 1 μM *N*-acetylserotonin, 0.2 μM mycophenolic acid, and 1 μM naloxone). The reactions were initiated by adding UDPGA, and the incubation continued for 60 min at 37 °C in a shaking water bath. The reactions were terminated by adding 50 μL of ice-cold acetonitrile containing internal standards (propofol glucuronide for chenodeoxycholic acid 24-acyl-β-glucuronide and mycophenolic acid glucuronide; and meloxicam for SN-38 glucuronide, trifluoperazine glucuronide, *N*-acetylserotonin β-d-glucuronide, and naloxone 3-β-D-glucuronide). The incubation mixtures were centrifuged at 13,000× *g* for 8 min at 4 °C. Next, 50 μL of each supernatant of A and B set was mixed, and aliquots (5 μL) were analyzed by LC–MS/MS. All assays were performed in triplicate, and the average values were used in calculations.

To measure the time-dependent inhibition, human liver microsomes were preincubated with the various concentrations of APINACA (final concentrations of 0.1–100 μM) and UDPGA for 30 min at 37 °C. Next, the reaction mixtures were incubated with UGT probe substrate cocktail sets for 60 min at 37 °C. The control reaction was performed by adding methanol instead of the test compounds.

The metabolites formed from the six UGT substrates were simultaneously separated using an Atlantis dC18 system (3 µm, 2.1 mm internal diameter × 100 mm) with a gradient elution of 5% acetonitrile in 0.1% formic acid (mobile phase A) and 95% acetonitrile in 0.1% formic acid (mobile phase B) at a flow rate of 0.3 mL/min: 10% mobile phase B for 1 min, 10% to 60% mobile phase B for 1 min, 60% to 95% mobile phase B for 1 min, 95% mobile phase B for 2 min, 95% to 10% mobile phase B for 0.1 min, and 10% mobile phase B for 2.9 min. The ESI source settings in both the positive and negative ion modes were as follows: gas temperature, 200 °C; gas flow, 14 L/min; nebulizer, 40 psi; sheath gas temperature, 380 °C; sheath gas flow, 11 L/min; capillary voltage, 4500 V; and nozzle voltage, 500 V. Each metabolite was quantified via selected reaction monitoring in the negative ion mode (chenodeoxycholic acid 24-acyl-β-glucuronide, *m/z* 567.1 → 391.2; mycophenolic acid glucuronide, *m/z* 495.0 → 319.0; propofol glucuronide (IS), *m/z* 353.0 → 177.0) and in the positive ion mode (SN-38 glucuronide, *m/z* 568.9 → 392.9; trifluoperazine glucuronide, *m/z* 583.9 → 407.9; *N*-acetylserotonin β-d-glucuronide, *m/z* 394.9 → 219.0; naloxone 3-β-D-glucuronide, *m/z* 503.9 → 309.9; meloxicam (IS), *m/z* 351.9 → 115.0).

### 4.4. Time-Dependent Inhibition of CYP3A4 Activity by APINACA in Human Liver Microsomes

The kinetic parameters for the time-dependent inhibition potency of APINACA against human liver microsomal CYP3A4 activity were evaluated. Ultrapooled human liver microsomes (1 mg/mL) were preincubated with various concentrations of APINACA (final concentrations of 1–40 μM) in 50 mM potassium phosphate buffer (pH 7.4) in the presence of NADPH. Aliquots (10 μL) of the preincubated mixtures were withdrawn 5, 10, 20, and 30 min after incubation was commenced and were added to other tubes containing 2 μM midazolam, 1 mM NADPH, 50 mM potassium phosphate buffer (pH 7.4), and 10 mM MgCl_2_ in 90-μL reaction mixtures. The second reactions were terminated after 10 min by adding 100 μL of ice cold methanol containing *d*_9_-1′-hydroxybufuralol. The incubation mixtures were centrifuged at 13,000× *g* for 8 min at 4 °C, and 50 μL of each supernatant was diluted with 50 μL of water. Aliquots (5 μL) of the diluted supernatants were analyzed by LC–MS/MS.

### 4.5. Enzyme Kinetic Analysis for the Inhibition of UGT1A9 by APINACA

To determine the enzyme kinetic parameters and mode of inhibition of UGT1A9 by APINACA, various concentrations of APINACA (final concentrations of 0–10 μM) and mycophenolic acid (final concentrations of 0.2–1.0 μM) as the UGT1A9 substrate were incubated with human liver microsomes (0.15 mg/mL), 10 mM MgCl_2_, 5 mM UGPGA, and 50 mM Tris buffer (pH 7.4) in a total volume of 100 μL for 60 min at 37 °C. The reaction was stopped by adding 100 μL of ice cold acetonitrile containing propofol glucuronide (internal standard), and the mixtures were centrifuged at 13,000× *g* for 4 min. Next, 50 μL of the supernatant was diluted with 50 μL of water, and aliquots (5 μL) were analyzed by LC–MS/MS.

### 4.6. Inhibitory Effect of APINACA on the Transport Activities of Efflux Transporters

LLC-PK1-MDR1 and LLC-PK1-mock cells were grown in tissue culture flasks in medium 199 supplemented with 8% FBS, 50 μg/mL of gentamycin, and 50 μg/mL of hygromycin. The cells were seeded onto filter membranes at a density of 2 × 10^5^ cells/well for 5 days with TEER values over 450 Ω·cm^2^. The B to A transport of 0.1 μM [3-H]digoxin in LLC-PK1-MDR1 cells was measured by adding 1.5 mL of HBSS containing APINACA (final concentrations of 0–100 μM) on the basal side and by adding 0.5 mL of HBSS without APINACA on the apical side of the insert using protocols identical to those described above. The B to A transport of 0.1 μM [3-H]digoxin in LLC-PK1-mock cells was also measured using the same protocol for comparison.

LLC-PK1-BCRP and -mock cells were grown in tissue culture flasks in DMEM supplemented with 10% FBS, 5 mM nonessential amino acids, and 100 U/mL of penicillin–streptomycin. The cells were seeded onto filter membranes at a density of 2 × 10^5^ cells/well for 5 days with TEER values over 300 Ω·cm^2^. The B to A transport of 0.1 μM [3-H]ES was measured by adding 1.5 mL of HBSS containing APINACA (final concentrations of 0–100 μM) on the basal side and by adding 0.5 mL of HBSS without APINACA on the apical side of the insert using protocols identical to those described above. The B to A transport of 0.1 μM [3-H]ES in LLC-PK1-mock cells was also measured using the same protocols for comparison.

Aliquots (100 μL) of transport samples were mixed with 200 μL of Optiphase cocktail solution (Perkin Elmer Inc.; Boston, MA, USA). The radioactivity of the probe substrate in the cells was measured using a liquid scintillation counter.

### 4.7. Inhibitory Effect of APINACA on the Transport Activities of Solute Carrier Transporters

HEK293 cells overexpressing OAT1, OAT3, OATP1B1, OATP1B3, OCT1, and OCT2 transporters and HEK293-mock cells were seeded in poly-d-lysine-coated 96-well plates at a density of 10^5^ cells/well and were cultured in DMEM supplemented with 10% FBS, 5 mM nonessential amino acids, and 2 mM sodium butyrate in a humidified atmosphere of 5% CO_2_ at 37 °C. For the experiments, the growth medium was discarded after 24 h, and the attached cells were washed with HBSS and preincubated for 10 min in HBSS at 37 °C.

To examine the effects of APINACA and typical inhibitors on transporter activity, the uptake of a probe substrate into HEK293 cells overexpressing the respective solute carrier transporters was measured in the presence of APINACA (final concentrations of 0–250 μM) or typical inhibitors for 5 min. The concentrations and probe substrates were selected as follows: 0.1 μM [3-H]MPP^+^ (for OCT1 and OCT2), 0.1 μM [3-H]PAH (for OAT1), 0.1 μM [3-H]ES (for OAT3 and OATP1B1), and 0.1 μM [3-H]EG (for OATP1B3). The uptake of the probe substrate into HEK293-mock cells was also measured using the same protocol for comparison. The typical inhibitors were selected as follows: cimetidine (0–250 μM) for OCT1 and OCT2, probenecid (0–250 μM) for OAT1 and OAT3, rifampin (0–250 μM) for OATP1B1 and OATP1B3, verapamil (0–250 μM) for P-gp, and sulfasalazine (0–250 μM) for BCRP.

The cells were then washed three times with 100 μL of ice cold HBSS immediately after placement of the plates on ice, and the cells were lysed with 50 μL of 10% sodium dodecyl sulfate and mixed with 150 μL of Optiphase cocktail solution (Perkin Elmer Inc.; Boston, MA, USA). The radioactivity of the probe substrates in the cells was measured using a liquid scintillation counter.

### 4.8. Data Analysis

The IC_50_ (the concentration of the inhibitor to show half-maximal inhibition) values were calculated using SigmaPlot ver. 12.5 (Systat Software, Inc.; San Jose, CA, USA). *K*_i_ (the inhibition constant), *k*_inact_ (the maximal rate of enzyme inactivation), and the mode of inhibition of CYP3A4 and UGT1A9 activities were determined using Enzyme Kinetics ver. 1.1 (Systat Software, Inc.).

## 5. Conclusions

The in vitro inhibitory effect of APINACA on eight major clinically important CYP and six UGT enzymes in ultrapooled human liver microsomes and on six solute carrier transporters and two efflux transporters using a transporter expression system was investigated for the first time to predict the drug interaction potential of APINACA via the modulation of drug-metabolizing enzymes and transporters. APINACA showed potent time-dependent inhibition of CYP3A4-mediated midazolam 1′-hydroxylation (*K_i_*, 4.5 μM) and noncompetitive inhibition of UGT1A9-mediated mycophenolic acid glucuronidation (*K_i_,* 5.9 µM), but did not show inhibition of other tested CYPs, UGTs, solute carrier transporters, and efflux transporters. These results suggest that it is necessary to evaluate the potential of APINACA as an in vivo cause of pharmacokinetic drug interactions via the inhibition of CYP3A4 and UGT1A9 enzymes.

## Figures and Tables

**Table 1:** Inhibitory potential of APINACA on eight major cytochrome P450 (CYP) enzyme activities with and without a 30-min preincubation in the presence of reduced β-nicotinamide adenine dinucleotide phosphate (NADPH) in ultrapooled human liver microsomes.

CYPs	Enzyme Activities	IC_50_ (μM)	
		No Preincubation	With Preincubation
1A2	Phenacetin *O*-deethylase	>100	>100
2A6	Coumarin 7-hydroxylase	>100	>100
2B6	Bupropion hydroxylase	>100	>100
2C8	Amodiaquine *N*-deethylase	>100	>100
2C9	Diclofenac 4′-hydroxylase	>100	85.0
2C19	[*S*]-Mephenytoin 4′-hydroxylase	>100	>100
2D6	Bufuralol 1′-hydroxylase	>100	>100
3A4	Midazolam 1′-hydroxylase	16.9	4.2

**Table 2:** Transport rate of the probe substrate in HEK293 cells overexpressing solute carrier transporters and LLC-PK1 cells overexpressing efflux transporters (*n* =3).

Cells	Transporters	Probe Substrate	Transport Rate (pmol/min) (mean ± SD)	Fold Increase
HEK293	Mock	0.1 μM Methyl-4-phenylpyridinium	0.60 ± 0.09	17.1
	OCT1		10.24 ± 0.98	
	Mock	0.1 μM Methyl-4-phenylpyridinium	0.66 ± 0.11	25.5
	OCT2		16.78 ± 0.43	
	Mock	0.1 μM para-aminohippuric acid	1.22 ± 0.22	17.6
	OAT1		21.49 ± 0.45	
	Mock	0.1 μM Estrone-3-sulfate	0.98 ± 0.20	15.3
	OAT3		14.96 ± 3.09	
	Mock	0.1 μM Estrone-3-sulfate	0.76 ± 0.04	16.5
	OATP1B1		12.58 ± 1.57	
	Mock	0.1 μM Estradiol-17β-d-glucuronide	0.17 ± 0.02	13.4
	OATP1B3		2.28 ± 0.08	
LLC-PK1	Mock	0.1 μM Digoxin	0.14 ± 0.02	7.3
	MDR1 (P-gp)		1.06 ± 0.07	
	Mock	0.1 μM Estrone-3-sulfate	0.44 ± 0.07	4.7
	BCRP		2.09 ± 0.12	

The data are expressed as the means ± SD from triplicate measurements.
